# Oblique convergence and strain partitioning in the outer deformation front of NE Himalaya

**DOI:** 10.1038/s41598-018-28774-3

**Published:** 2018-07-12

**Authors:** Dibyashakti Panda, Bhaskar Kundu, M. Santosh

**Affiliations:** 1 Department of Earth and Atmospheric Sciences, NIT Rourkela, Rourkela, 769008 India; 2 School of Earth Science and Resources, China University of Geosciences, Beijing, China; 3 Department of Earth Sciences, University of Adelaide, SA, 5005 Australia; 4 State Key Laboratory of Continental Dynamics, Department of Geology, Northwest University, Xi’an, 710069 China

## Abstract

Himalayan-Tibetan orogeny has considered as a natural black box in the context of geodynamic evolution and tectonic complexity. The eastward extrusion model of Tibetan crust contradicts with the oblique convergence model in the NE-Himalaya (Bhutan/Arunachal region), where the overall convergence rate accommodated in the Himalaya is about 20–25% less than that in the neighbouring central Himalaya and Eastern Himalayan syntaxis (EHS). We propose that instead of partitioning in the backarc, the NE-Himalaya has developed an active sliver along the Assam-Brahmaputra valley in the outer deformation front, in order to accommodate the deficiency in long-term plate convergence between Himalaya and southern Tibet. We argue that the strong eastward extrusion of Tibetan crust along NE-Himalaya is the main driving force for the unusual development of the Assam-Brahmaputra sliver. This new hypothesis can explain active convergence along EHS, low convergence and subdued topography in Bhutan and Arunachal Himalaya, kinematic and space-problem of Indo-Burmese wedge, and finally solves the contradiction between Tibetan extrusion and oblique convergence model of the HimalayanTibetan orogeny.

## Introduction

The dynamic topography of the Earth’s surface, particularly those along continental margins, has been linked to mantle processes in recent studies^1,2^. The spectacular topographic features and tectonic history of Himalayan-Tibetan orogen has received global attention in terms of continental collision and related geodynamic processes, seismic hazards, and Asian climatic patterns, among other themes^3,4^. Among the geodynamic implications, two popular models have been in focus during the past decades: (a) ductile “glacier-like flow” of the Tibetan lower or upper crust and associated distribution in deformation^5–7^; and (b) interference of rigid lithospheric blocks and localised deformation along active faults^8,9^.

The oblique convergence model which describes the deformation between Himalaya and southern Tibet along the frontal-arc^8^, and the lateral extrusion model which mainly considers deformation of Tibet^8^, appear to be mutually contradictory in view of the tectonics and distributed deformation (or slip partitioning) in the NE-Himalaya^8^. It has been proposed that in the NW-Himalaya, the motion between India and southern Tibet is oblique with respect to the structural trend, which is partitioned between right-lateral strike-slip motion on the Karakorum fault system in the back arc, and slightly oblique motion in the Kashmir Himalaya in the frontal arc, leading to the formation of the NW-Himalayan sliver^10^. However, in the adjoining Garhwal-Kumaun and Nepal Himalaya, there is no partitioning, and the entire convergence of 18–20 mm/yr occurs in arc-normal manner^11,12^.

**Figure 1:**
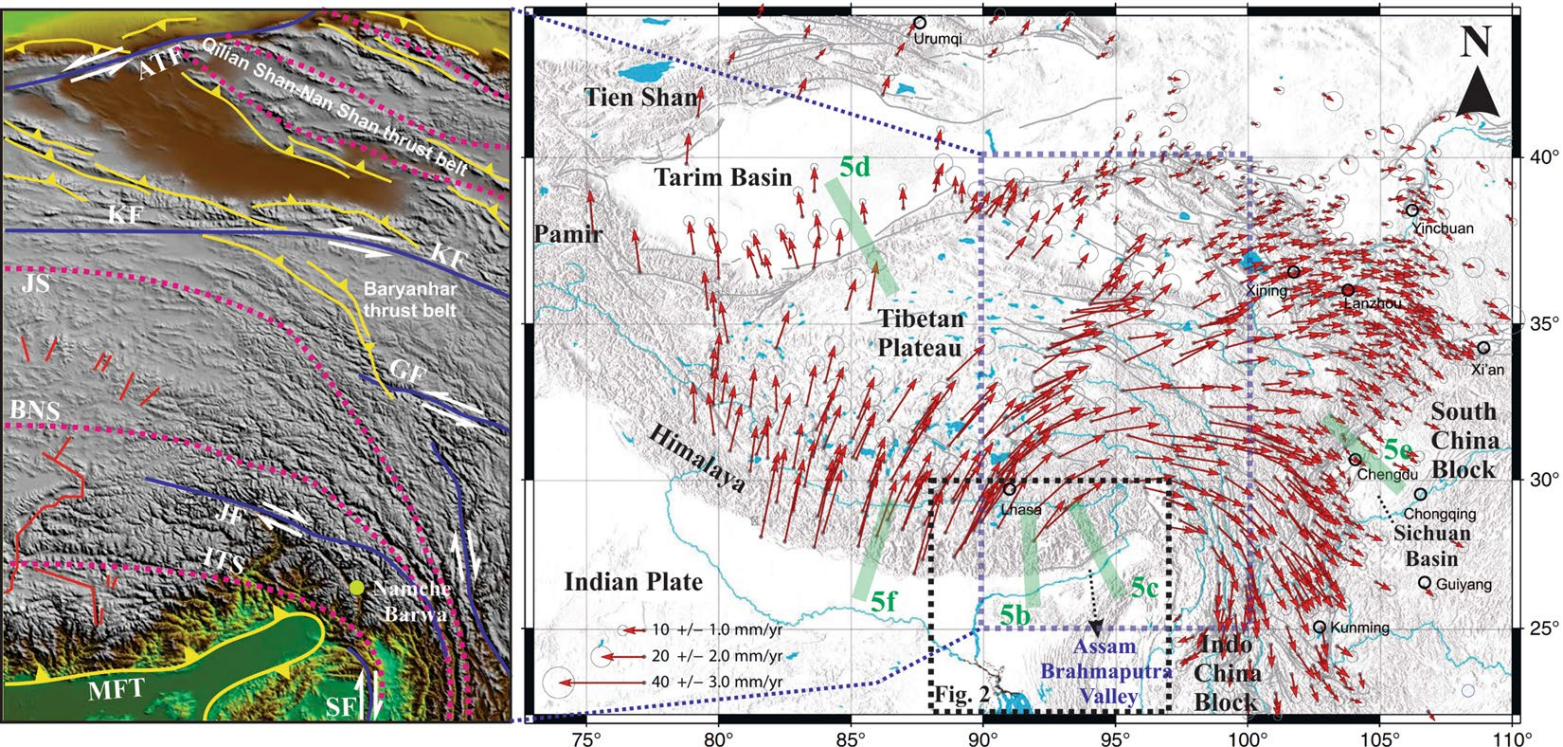
Eastward extrusion of the Tibetan crust. Horizontal GPS velocities of crustal motion relative to stable Eurasia^7^, with is 95% confidence. The thick gray solid lines indicate active faults in Holocene. Green lines represent axes of swath profiles for topographic relief for different margins of plateau, considered in channel flow model in Fig. 5. Detailed tectonic features are represented in the left panel (taken from *Yin and Taylor*,^25^). Suture zone, strike slip faults, thrusts faults and normal faults are marked by pink dotted line, blue line, yellow line and red line respectively. ATF- Altyn Tagh fault, KF-Kunlun fault, GF-Ganzi fault, JS-Jinsha suture, BNS-Bangong Nujiang suture, JF-Jiali fault, ITS-Indus Tsangpo suture, MFT-Main Frontal Thrust, SF-Sagaing fault. **(**Figure was generated using Generic Mapping Tools (version 5.2.1; URL: http//gmt.soest.hawaii.edu/) and Global Mapper application (version 17.0.5).

In the NE-Himalaya (composed of Bhutan and Arunachal), the motion between India and southern Tibet is again oblique with respect to structural trend of the arc (Fig. 1). In order to accommodate such oblique plate motion, another sliver in the NE-Himalaya with prominent left-lateral strike-slip fault system in the back arc and to the northwest of the EHS is required. However, geomorphic offsets, geodetic estimates and the available earthquake data do not support the presence of any active left-lateral fault system in the region^13^.

This raises question on applicability of the oblique convergence model in the NE-Himalaya. Here we address this issue and propose that instead of partitioning in the back arc, an active sliver in NE Himalaya has developed along the Assam-Brahmaputra valley in the outer deformation front which is accommodating the oblique motion through distributed deformation. The strain partitioning in the outer deformation front of Himalayan region has not been well evaluated in previous studies. Here we take into account the Assam-Brahmaputra valley sliver in the outer deformation front of NE Himalaya, in order to explain the complex geodynamic process. We also propose that the strong eastward flow of Tibetan crust around the EHS, investigated using different methods in different time scale^14–33^, is mainly responsible for such unusual development of a sliver in the outer deformation front of the NE-Himalaya. Based on the GPS observations, we address the oblique convergence and outer deformation-front strain partitioning process along the Assam-Brahmaputra valley adjacent to NE-Himalaya.

## Assam-Brahmaputra valley sliver in the outer deformation front along NE-Himalaya

The GPS derived convergence rates along the Himalayan arc between the Indian plate and southern Tibet have been reported by several authors^10–12,34–37^. The estimate varies from ~13 mm/yr in Kashmir^10,35^ to 18–20 mm/yr in the central Himalaya^11,12,34^ to 15–16 mm/yr in Bhutan Himalaya^37^ and 19–20 mm/yr in the Eastern Himalayan syntaxis^38^. To accommodate the oblique motion between India and southern Tibet in the NW-Himalaya, the convergence is partitioned into right-lateral strike-slip motion on the Karakorum fault system in the back arc and oblique motion in the Kashmir Himalaya along frontal arc, forming NW-Himalayan sliver^10^. On the other hand, it has argued that in the NE-Himalaya, the Brahmaputra valley has broken apart from the Indian plate and has rotated in a clockwise direction, resulting in the lowering of relative plate convergence rate across the NE-Himalaya^37^.

In view of relative contradiction in oblique convergence and eastward extrusion model of Tibet, we propose here a new hypothesis for the NE Himalaya that is reasonably more compatible in the context of regional tectonics and deformation. We argue that the uncertainties in the GPS site velocities (~3.5 mm/yr) and poor spatial distribution of the data points from the Arunachal and Assam-Brahmaputra valley region appear to be inconsistent for demarcation of micro-block’s boundaries and associated deformation patterns. Also, the differential rotation is difficult to constrain (or distinguish) the variation in slip-rate along the strike of the fault system.

**Figure 2:**
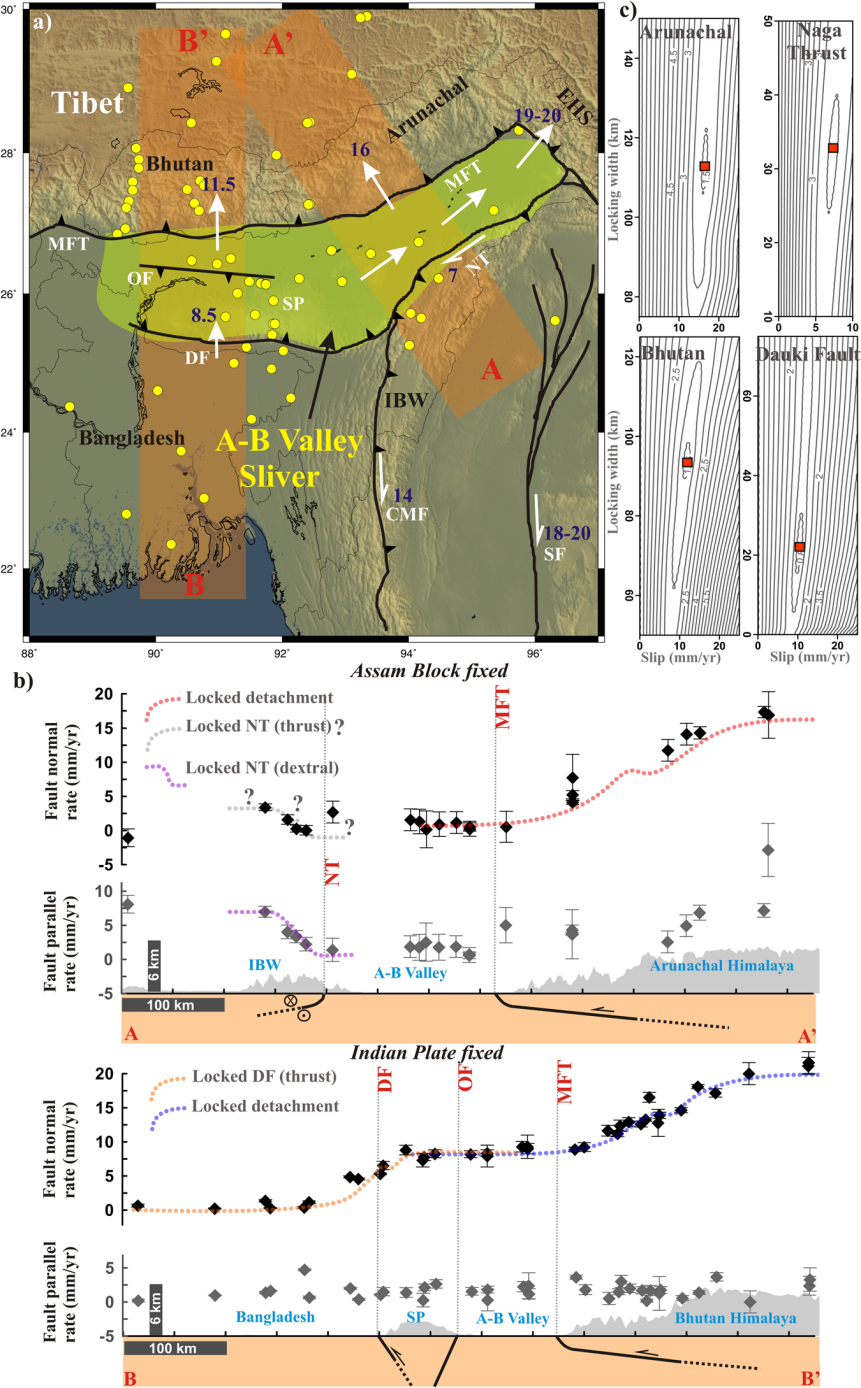
Distributed deformation across NE-Himalaya. (**a**) Regional tectonics of the Indo-Burmese Arc, Assam-Brahmaputra Valley sliver and NE-Himalaya. White arrows show the distributed slip across different fault systems around A-B valley. Yellow dots show the cGPS sites used in the study. (**b**) Fault normal and fault parallel rate (velocity) are considered across two swath profiles A-A′ and B-B′ respectively. (**c**) Represents error analysis using weighted RMS error using grid search method with varying slip and width of locked zone of the respective faults. The red square indicates the least error region. Note A-B valley participates in NE Himalayan strain accumulation process. IBW-Indo Burmese wedge, MFT-Main frontal thrust, EHS-Eastern Himalayan Syntaxis, A-B valley-Assam Brahmaputra valley, NT-Naga thrust, DF-Dauki fault, OF-Oldham fault, SP-Shillong Plateau, SP-Sagaing Fault, CMF-Churachandpur-Mao Fault. Figure (**a**) was generated using Generic Mapping Tools (version 5.2.1; URL: http//gmt.soest.hawaii.edu/).

We know that, a rigorous alignment of the different velocity fields currently available for the investigated region in recent literature, can be performed by solving for the Helmert transformation parameters that minimize the RMS of differences between velocities at common sites^39^. However, lacking of enough common sites among some of the available velocity fields, we estimated an unique velocity field for Bhutan, Arunachal, Assam, Tibet, Bangladesh, Shillong and Indo-Burmese wedge^7,11,34,37,40–44^, by transforming all available velocity field into the ITRF2008^45^ using the National Geodetic Survey’s horizontal time dependent positioning tool (https://www.ngs.noaa.gov/TOOLS/Htdp/Htdp.shtml). Finally, all the measurements were transformed into two relative reference frames, with India-fixed^34^ and the Assam block fixed^37^ respectively for two transects B-B’ and A-A’ (shown in Fig. 2a). We consider that the relative reference frame is a better choice for characterising strain accumulation process across active faults along plate boundary/plate-interior domains. To quantify it further, we resolved the relative reference frame bearing velocities into fault parallel and fault normal components, considering their local fault-normal distance and regional strike, considering reliable data points into two transects across A-A′ and B-B’ profiles respectively (marked in Fig. 2a). We consider that two transects (A-A′ and B-B′) are nearly perpendicular with the regional strike of the faults in Assam-Brahmaputra Valley and adjacent NE-Himalaya (Fig. 2a). Finally, we have adopted elastic dislocation model in a half-space^46^, to calculate fault slip-rate and locking width/depth. Applying a grid search approach which minimises the misfit between the observed and simulated velocity, we predict the width of locked zone along with the slip-rate of different fault segments along these two transects (Fig. 2c, Table 1, see Supplementary file).

We present velocity distribution across A-A′ profile considering the Assam block as fixed reference frame^37^. However, the velocity distribution across B-B’ profile is represented using India-fixed reference frame^34^ as shown in Fig. 2b. We identify that ~7 mm/yr dextral strike-slip motion is absorbed by the Naga thrust. However, there are uncertainties in fault normal velocity distributions that restrict further comments on this aspect. Moreover, available earthquake focal mechanism data are also consistent with the dextral motion of Naga thrust^47^. Further north in the Arunachal Himalaya, deformation front of Himalaya (MFT) absorbs about ~16 mm/yr arc normal convergence, although uncertainties in fault parallel velocity limit further interpretations (Fig. 2b). In contrast, we found that the deformation front of Bhutan Himalaya is accommodating ~11.5 mm/yr arc normal convergence and the Dauki fault takes about ~8.5 mm/yr fault normal motion. However, we do not observe any significant amount of fault parallel motion across that profile (Fig. 2b). Therefore, it appears that overall convergence rate is about 20–25% lesser in NE-Himalaya (i.e., in the Bhutan Himalaya 25% lesser and in the Arunachal Himalaya 20% lesser) than in the neighbouring central Himalaya and Eastern Himalayan syntaxis (EHS). It indicates that Naga thrust and Dauki fault are definitely participating in NE-Himalayan strain budget via distributed deformation along Assam-Brahmaputra valley.

We propose that instead of partitioning in the back arc, the NE-Himalaya has developed an active sliver along the Assam-Brahmaputra valley in the outer deformation front of MFT which accommodates the remaining 20–25% deficiency in motion through distributed deformation along the Dauki Fault and Naga thrust. Strong eastward extrusion of Tibetan crust along NE-Himalaya and adjacent EHS is mainly responsible for such unusual development of sliver tectonics in the outer deformation front of the NE-Himalaya (Fig. 2). Moreover, a recent shear wave anisotropy study in the Eastern Himalaya, Indo-Burmese arc and adjoining regions also compliment with our proposed sliver tectonics in the outer deformation front^48^.

Existence of the Assam-Brahmaputra valley sliver in the outer deformation front of the NE-Himalaya as proposed in this study can explain a number of processes such as (Fig. 2a): (i) active convergence along EHS, (ii) geometrical and spatial existence of the Assam-Brahmaputra valley in the region, (iii) low convergence and subdued topography in Bhutan and Arunachal Himalaya, (iv) kinematic and space-problem of Indo-Burmese wedge and finally (v) solves the contradiction between Tibetan extrusion and oblique convergence model of Himalayan Tibetan orogeny.

## Concluding Discussion

### Shillong Plateau Assam-Brahmaputra valley sliver and Indo-Burmese arc interaction: a zipper junction analogy

Geodynamic evolution of the Indo-Burmese arc associated fold-and-thrust belt structure is significantly influenced by the interaction with the Shillong Plateau and Assam-Brahmaputra valley formation process^49,50^. It has argued that rapid uplift of Shillong Plateau and westward encroachment of the outer Indo-Burmese wedge are closely related^49^. *Maurin and Rangin*^49^, suggested that during the time range from Late Eocene to Early Oligocene, the Shillong Plateau was significantly uplifted, that facilitated the rapid westward propagation of the outer wedge. Further, it has argued that the systematic westward overprinting from thin-skinned tectonics to thick-skinned tectonics was a manifestation of rapid onset of westward wedge propagation. Recently, using seismic, sedimentological, stratigraphic constrains from the Surma Basin and palaeodrainage analyses of the Brahmaputra river, *Najman et al*^50^., upholds the westward wedge propagation hypothesis.

**Figure 3:**
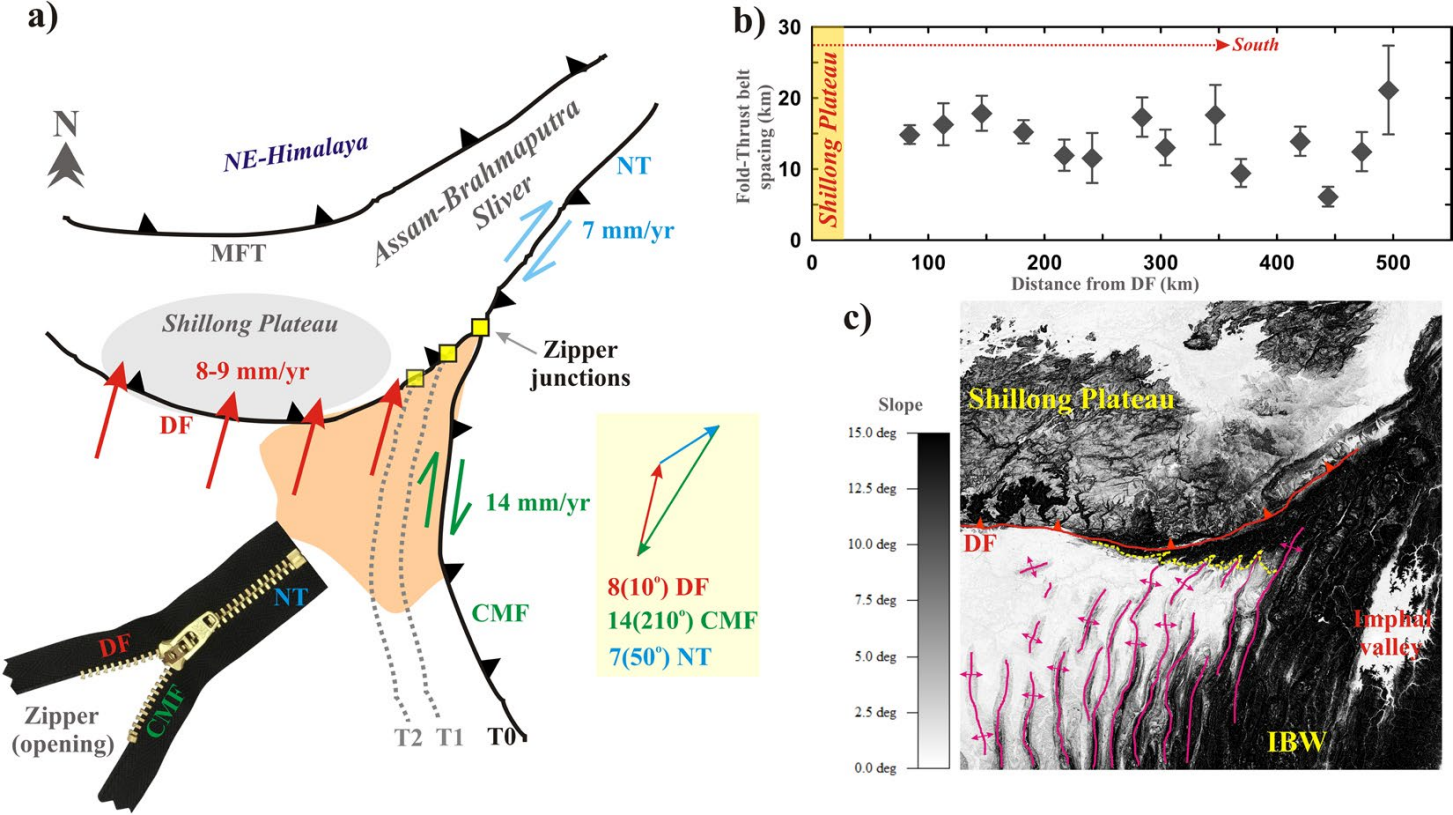
Assam-Brahmaputra valley sliver and Indo-Burmese wedge interaction. (**a**) An opening zipper junction analogy. T0, T1, T2 represents systematically young to older time and corresponding geometry of the IBW (grey dashed lines) and zipper junctions (yellow box). Plate motion representation at the Zipper junction is also shown. (**b**) Variation in fold-and thrust belt spacing with increasing southward distance from the Dauki Fault (DF). (**c**) Abutting fold-and thrust belt beneath the Shillong Plateau (in slope shaded map view). NT-Naga thrust, DF-Dauki fault, CMF-Churachandpur-Mao Fault, MFT-Main Frontal Thrust, IBW-Indo-Burmese Wedge. Global Mapper application (version 17.0.5) has been used to visualize slope shaded map in Figure (**c**).

Further, existence of Shillong Plateau/Assam-Brahmaputra valley in north of the Indo-Burmese arc, a hyper-oblique to strike-slip plate boundary region may acts as a structural buttress for the outer wedge. Therefore, as a consequence westward wedge propagation and systematic change in fold-and-thrust belt spacing are expected in order to resolve space problem in the region. However, fold-and-thrust belt spacing of the outer wedge does not show any systematic variation with respect to increasing distance further south from the Shillong Plateau (Fig. 3b,c). Moreover, geometrical and kinematic point of view the Shillong Plateau/Assam-Brahmaputra valley sliver and Indo-Burmese arc interaction are still elusive.

It has been proposed that segments of active faults with opposing slip sense are rarely offsets each other in reality, but often merge into single fault system, resulting in geometrical and kinematic problems^51,52^. The space and kinematic problems can be resolved by lengthening the merged faults (zipping) or splitting it (unzipping), named as “Zipper junctions”^51,52^. We proposed an opening “Zipper junctions” analogy to explain Shillong Plateau/Assam-Brahmaputra valley sliver and Indo-Burmese wedge interaction process (Fig. 3a), where south-eastward dipping Naga thrust, northward dipping Dauki fault and eastward steeply dipping Churachandpur-Mao Fault representing three arms of the junction (Fig. 3a). This opening “Zipper junctions” not only solves the issue of space problem in the outer wedge of Indo-Burmese arc, but also resolve kinematic, having a slip rate equal to the vector sum of the geodetic slip rates on the merging faults (i.e., Naga thrust, Dauki fault and Churachandpur-Mao Fault). Therefore, it appears that “Zipper junctions” analogy and Shillong Plateau/Assam-Brahmaputra valley sliver tectonics are mutually complimented with each other (Fig. 3).

### Backarc vs. outer deformation fronts slip partitioning

**Figure 4:**
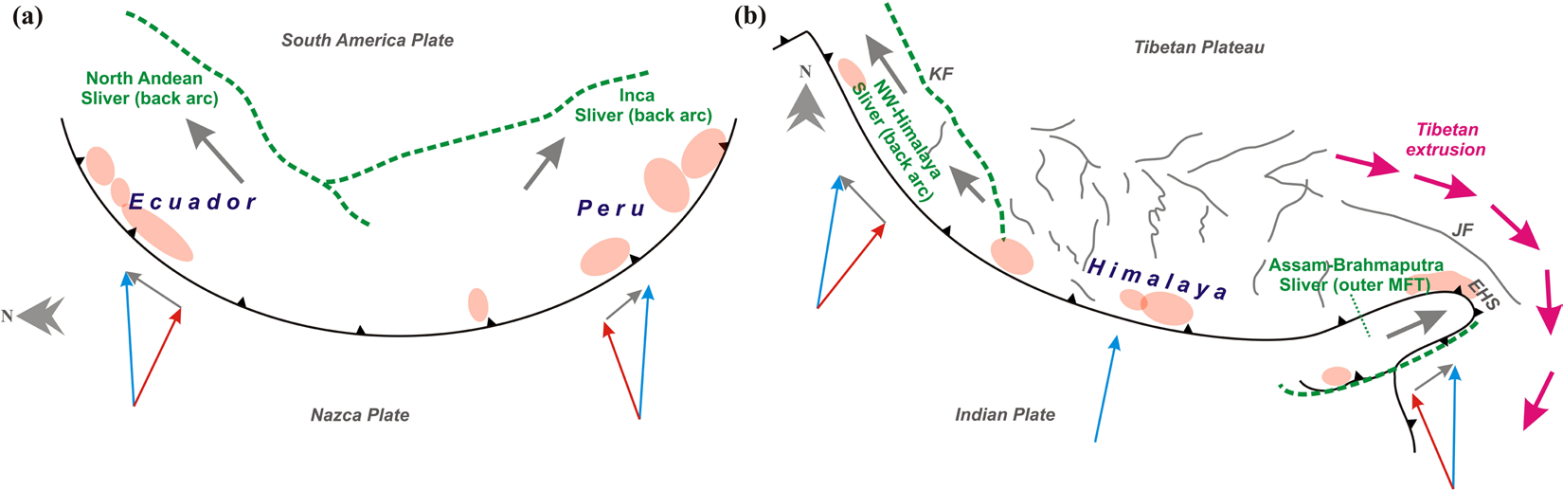
Backarc vs. outer deformation-front stain partition. Distributed deformation in two different convergent plate margins: (**a**) South America (Ecuador and Peru) and (**b**) Himalayan arc. Note eastward flow of Tibetan crust influences outer deformation-front stain (or slip) partitioning along Assam-Brahmaputra valley region in NE-Himalaya. Red, grey and blue vectors represent arc normal convergence, arc parallel sliver motion and resultant relative plate convergences respectively. KF-Karakorum Fault, JF-Jiali Fault, EHS- Eastern Himalayan Syntaxis. Figure (**a**) and (**b**) were generated using Corel Draw (version 13) graphical application (URL: https://www.coreldraw.com/en).

The term “slip partitioning” is suggested to describe the oblique motion along tectonic boundaries which is accommodated into two or more faults systems through different mechanisms^53^. The detachment of forearc blocks from the overriding plate and development of backarc sliver block during oblique convergence has been documented globally^53,54^. For example, along the orogenic boundary of the South America, the relatively plate convergence of the Nazca Plate at ~60–70 mm/yr, contributes to the deformation of the overriding continents from Colombia, Ecuador, Peru and Chile, through complex sliver plate motion of discreet domains^55^. Recent geodetic measurements suggest that two largest continental slivers (North Andean Sliver and Inca Sliver), which forms in the backarc domain are the result of the obliquity of relative plate convergence^55^ (Fig. 4a).

However, slip partitioning in outer trench (or deformation front) is rare and *Ishii et al*^56^., introduced this to explain the 11 April, 2012 Indian Ocean Earthquake of M 8.6 in the diffused plate boundary region of the Wharton basin, where the Indo-Australian plate is obliquely subducted beneath the Sunda plate. It has been argued that internally deformable blocks associated with pre-existing faults on the outer deformation fronts (or trench) are essential conditions for the outer trench (or deformation front) slip partitioning. Similarly, the outer deformation front of MFT in the NE-Himalaya, the Assam-Brahmaputra valley region, also satisfies the essential condition for the slip partitioning (Fig. 4b).

### Strong vs. weak topographic margins: implication on Himalayan seismic hazard

**Figure 5:**
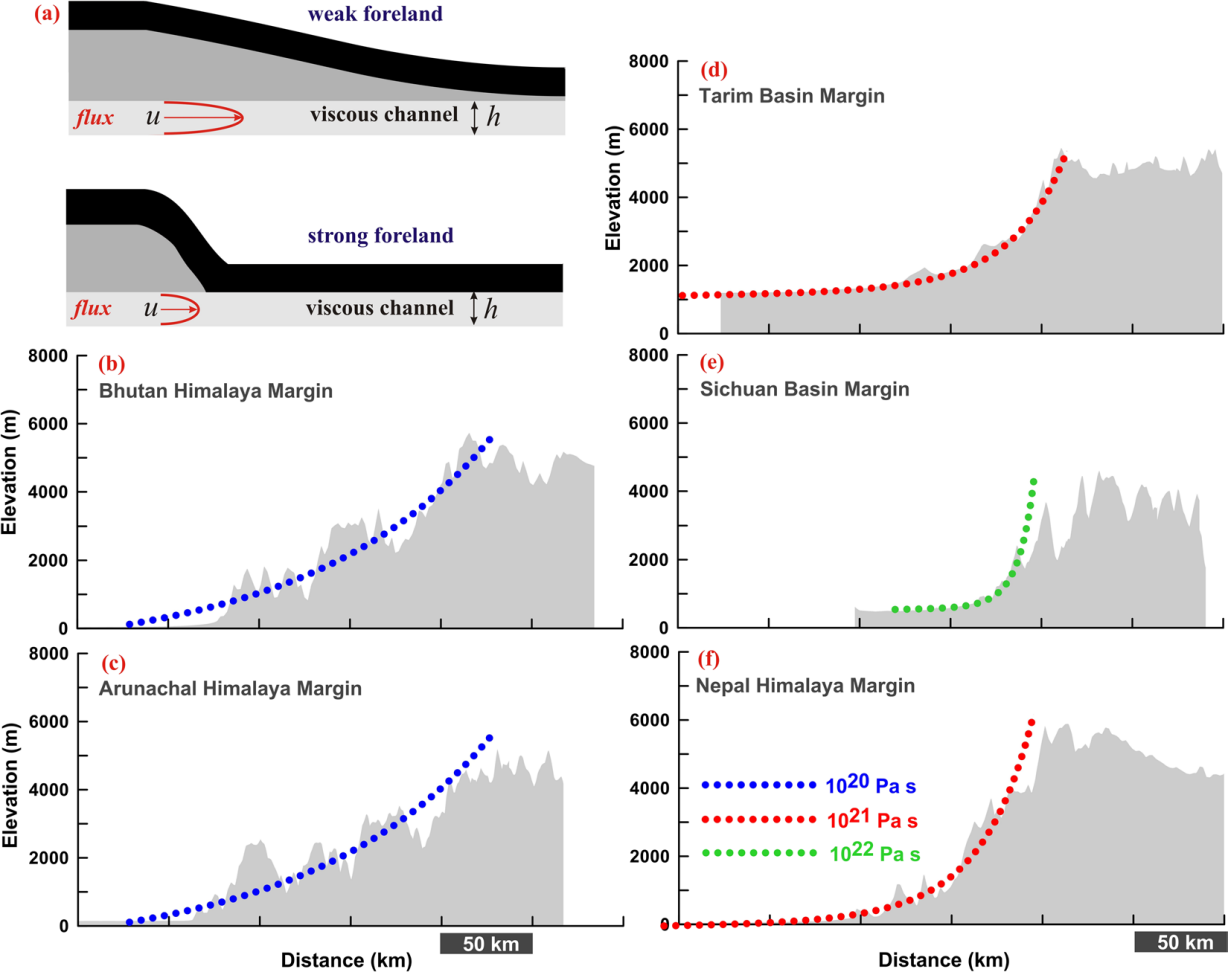
Viscosity and topographic margins. (**a**) Schematic representation of viscous channel flow model^30^. Material is allowed to flux into a viscous channel of uniform thickness ‘h’ with specific viscosity for run time of 20–25 Ma, so that the elevation is reached at the edge of the plateau. Viscosity of the channel material in foreland determines the regional topographic slope. (**b–f**) Model prediction vs. maximum topographic profiles for different margins of plateau (marked in Fig. 1). Assam Brahmaputra valley region adjacent to Bhutan and Arunachal Himalaya margins behaves as a relatively weak foreland with respect to the Sichuan Basin or Nepal Himalaya margin. Global Mapper application (version 17.0.5) has been used to visualize the topographic variation.

**Figure 6:**
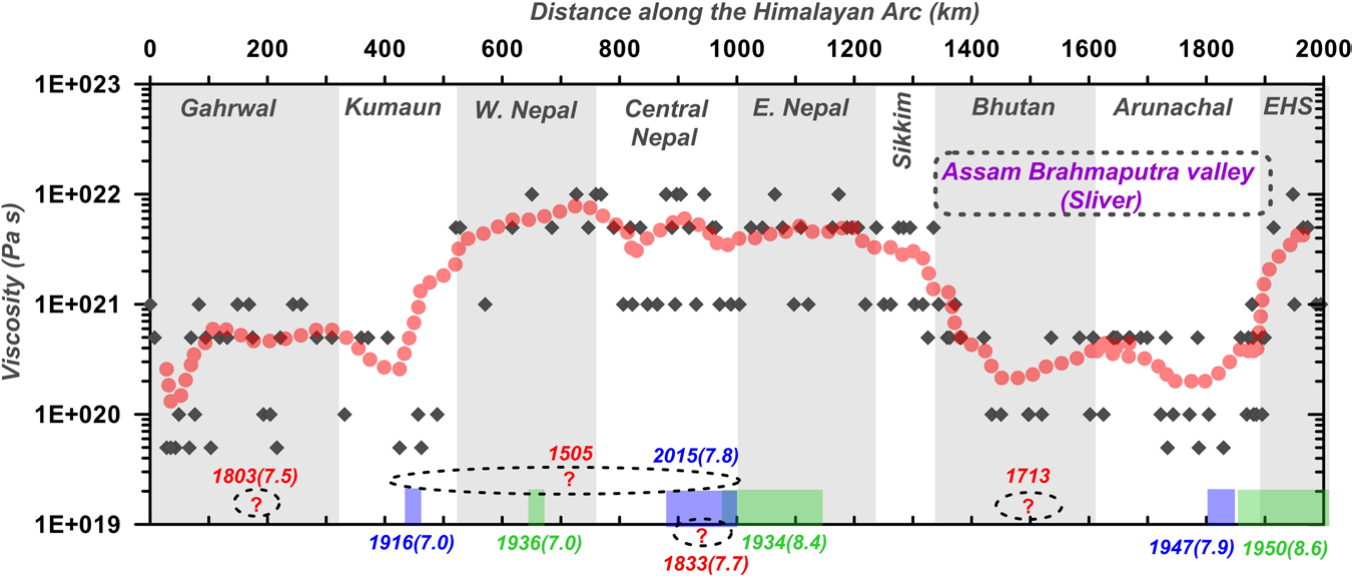
Predicted variation in viscosity of the channel material in foreland determined from the regional topographic slope along the Himalayan Arc. Note that the Assam Brahmaputra valley region adjacent to Bhutan and Arunachal Himalaya, Gahrwal and some portion of Kumaun Himalaya margins behave as relatively weak foreland with respect to Nepal Himalaya and EHS margin. Himalayan rupture zones of significant historical earthquakes are marked^11,12,37,59^. Note weak foreland segments are lacking significant historical earthquakes. We have considering longitude 77.5°E as zero. Black diamond’s represents actual predicted viscosity from the regional topographic slope across 150 transects along the Himalayan arc and bold red curve represents running average variation in predicted viscosity along the arc. Global Mapper application (version 17.0.5) has been used to visualize the topographic variation.

Considering the Newtonian channel flow model through lower crust of Tibet, *Clark and Royden*^30^ has explored the rheological property (see the Materials and Methods), by quantifying geometry of the topographic margins (Fig. 5a). Modelled topographic margin in the Bhutan-Arunachal Himalaya margins adjacent to the Assam-Brahmaputra valley (Fig. 5b,c), yield an excellent fit with the observed topography for a lower crustal viscosity of about 10^20^ Pa s. However the central Nepal, Sichuan basin, and Tarim basin margins show excellent fit with relatively higher crustal viscosity range of about 10^22^-10^21^ Pa s (Fig. 5d–f). Using this approach, we have predicted variation in viscosity of the channel material along the Himalayan arc starting from Gahrwal-Kumaun to EHS (Fig. 6). It appears that the Assam-Brahmaputra valley region adjacent to Bhutan-Arunachal Himalaya and Gahrwal-Kumaun Himalaya topographic margins are significantly weaker as compared to the surrounding Nepal and EHS margins. Further, gravity data from Bhutan Himalaya suggests decoupled lithospheric layers leading to an eastward decrease in flexural rigidity of Indian plate from 10^24^ Nm to 5 × 10^22^ Nm in the Nepal and Bhutan Himalaya respectively^57^.

We argue that, such a weaker topographic margin across Bhutan/Arunachal Himalaya adjacent to the Assam-Brahmaputra valley region appears to be suitable for the slip-partitioning in outer deformation front. It also implies that these weaker margins are well compatible with the internal deformation process. However, Nepal and EHS margins are much more strong and hence, vulnerable for interseismic strain build up and eventually release process by hosting great devastating earthquakes (Fig. 6). In fact, rupture zones of significant historical and recent earthquakes along the Himalayan arc appear to be indirect proxy for the along-arc rheological variation.

## Materials and Methods

### Modelling foreland topographic margins

Considering Newtonian channel flow model through lower crust of Tibet, *Clark and Royden*^30^ explored the rheological properties by quantifying geometry of the topographic margins (Fig. 5a). They proposed a model of ductile flow in the lower crust of uniform thickness in which the crustal material is allowed to flow in response to pressure gradients through a uniform channel thickness (h) (Fig. 5a). For Poiseuille flow with zero velocity at the top and at the bottom of the channel, the velocity (u) of crustal material in the channel as a function of viscosity (μ), lateral pressure gradient (dp/dx), and depth (y) is represented as^58^:

1 $$u=\frac{1}{2\mu }\cdot \frac{dp}{dx}\,({y}^{2}-yh)$$

The flux (u) of the material in the channel can be expressed by integrating the velocity of the material over the channel height (h),

2 $$u={\int }_{0}^{h}u\,dy=-\frac{1}{12\mu }\cdot \frac{dp}{dx}[{h}^{3}]$$

Again, it can be related to changes in crustal thickness (c) over time:

3 $$\frac{dc}{dt}=-\frac{du}{dx}=\frac{1}{12}\cdot {h}^{3}\cdot \frac{d}{dx}(\frac{1}{\mu }.\frac{dp}{dx})$$

Further, considering $p={\rho }_{c}.g.{T}_{(x)}$ we get:

4 $$\frac{dc}{dt}=\frac{1}{12}\cdot {h}^{3}\cdot \frac{d}{dx}[\frac{1}{\mu }\cdot \frac{d}{dx}({\rho }_{c}\cdot g\cdot {T}_{(x)})]\frac{dc}{dt}=\,\frac{1}{12}\cdot {h}^{3}\cdot {\rho }_{c}\cdot g\cdot \frac{d}{dx}(\frac{1}{\mu }.\,\frac{dT}{dx})$$

It has been assumed that the lateral pressure gradient in the channel is a function of topography, where ρ_c_ is the average crustal density (2800 kg/m^3^), g is the gravitational acceleration, and T_(x)_ is the topographic elevation. By considering topographic wavelengths that are long compared to the flexural wavelength of the crust (according to concept of Airy isostatic equilibrium^58^), changes in topographic relief can be linearly related to variations in crustal thickness. Therefore, the change in topographic elevations over time as a result of flux of crustal material in the lower crust can be expressed by:

5 $$\frac{dT}{dt}=\frac{{\rho }_{m}-{\rho }_{c}}{{\rho }_{m}}[\frac{1}{12}\cdot {h}^{3}\cdot {\rho }_{c}\cdot g\cdot \frac{d}{dx}(\frac{1}{\mu }\cdot \frac{dT}{dx})]$$

where, ρ_m_ = mantle density = 3300 kg/m^3^.

Using the above concept, we generated swath topographic profiles across Himalaya and surrounding margins of Tibet (Fig. 5) using topographic data from SRTM of digital elevation, produced by NASA (http://www.cgiar-csi.org). We assumed a spatially uniform lower crustal viscosity in the 15 km lower crustal channel. Flux rate was considered to allow the plateau margin of average 5 km elevation to be produced over 20–25 Ma^30^. Model topographic profile in the Tarim, Sichuan and Nepal Himalaya fault margins, yields an excellent fit with the topography margins for a higher crustal viscosity of about 10^21^ to 10^22^ Pa s, however in the Bhutan and Arunachal Himalaya segments a good fit is obtained with lower viscosity of about 10^20^ Pa s (Fig. 5b–f). Using same concept we have predicted variation in viscosity of the channel material in foreland across the Himalayan Arc (Fig. 6). Further, Global Mapper application (version 17.0.5) has been used to visualize the topographic variation and profiles in this analysis.

## Electronic supplementary material

Supplementary Information

## References

[1] Colli, L., Ghelicchkhan, S., Bungle, H.-P. & Oeser, J. Retrodictions of Mid Paleogene mantle flow and dynamic topography in the Atlantic region from compressible high resolution adjoint mantle convection models: Sensitivity to deep mantle viscosity and tomographic input model. *Gondwana Research*, 10.1016/j.gr.2017.04.027 (2017).

[2] Muller, R. D., Hassan, R., Gurnis, M., Flament, N. & Williams, S. E. Dynamic topography of passive continental margins and their hinterlands since the Cretaceous. *Gondwana Research*, 10.1016/j.gr.2017.04.028 (2017).

[3] Chatterjee S, Goswami A, Scotese CR (2013). The longest voyage: Tectonic, magmatic, and paleoclimatic evolution of the Indian plate during its northward flight from Gondwana to Asia. Gondwana Research.

[4] Zhang Z, Ding L, Zhao Z, Santosh M (2017). Tectonic evolution and dynamics of the Tibetan Plateau. Gondwana Research.

[5] Thatcher W (2007). Microplate model for the present-day deformation of Tibet. J. Geophys Res..

[6] Klemperer SL (2013). Mantle fluids in the Karakoram fault: Helium isotope evidence. Earth Planet Sci Lett..

[7] Liang S (2013). Three-dimensional velocity field of present-day crustal motion of the Tibetan Plateau derived from GPS measurements. J. Geophys Res..

[8] Styron RH, Taylor MH, Murphy MA (2011). Oblique convergence, arc-parallel extension, and the role of strike-slip faulting in the high Himalaya. Geosphere.

[9] Murphy MA (2013). Limit of strain partitioning in the Himalaya marked by large earthquakes in western Nepal. Nat Geosci..

[10] Kundu B, Yadav RK, Bali BS, Chowdhury S, Gahalaut VK (2014). Oblique convergence and slip partitioning in the NW Himalaya: Implications from GPS measurements. Tectonics.

[11] Ader T (2012). Convergence rate across the Nepal Himalaya and interseismic coupling on the main Himalayan thrust: Implications for seismic hazard. J. Geophys Res..

[12] Stevens VL, Avouac J-P (2015). Interseismic coupling on themain Himalayan thrust. Geophys Res Lett..

[13] Woerd JV, Leloup P-H, Zeng JL, Lscassin R, Tapponnier P (2009). A comment on “Orogen-parallel, active left-slip faults in the eastern Himalaya: Implications for the growth mechanism of the Himalayan arc” by Li and Yin. Earth Planet Sci Lett..

[14] Zhang C, Cao J, Shi Y (2009). Studying the viscosity of lower crust of Qinghai–Tibet Plateau according to post-seismic deformation. Science in China.

[15] Zhang Z (2009). Crustal structure across Longmenshan fault belt from passive source seismic profiling. Geophys Res Lett..

[16] Ryder I, Bürgmann R, Sun J (2010). Tandem afterslip on connected fault planes following the 2008 Nima-Gaize (Tibet) earthquake. J Geophys Res..

[17] Ryder I, Bürgmann R, Pollitz F (2011). Lower crustal relaxation beneath the Tibetan Plateau and Qaidam Basin following the 2001 Kokoxili earthquake. Geophys J Int..

[18] Yamasaki T, Houseman GA (2012). The crustal viscosity gradient measured from post-seismic deformation: a case study of the 1997 Manyi (Tibet) earthquake. Earth Planet Sci Lett..

[19] Wen Y, Li Z, Xu C, Ryder I, Bürgmann R (2012). Postseismic motion after the 2001 Mw7.8 Kokoxili earthquake in Tibet observed by InSAR timeseries. J Geophys Res..

[20] Huang M-H, Bürgman R, Freed AM (2014). Probing the lithospheric rheology across the eastern margin of the Tibetan Plateau. Earth Planet Sci Lett..

[21] DeVries PMR, Meade BJ (2013). Earthquake cycle deformation in the Tibetan plateau with a weak mid-crustal layer. J Geophys Res..

[22] Hilley GE (2005). Bayesian inference of plastosphere viscosities near the Kunlun Fault, northern Tibet. Geophys Res Lett..

[23] Hilley GE (2009). Earthquake-cycle deformation and fault slip rates in northern Tibet. Geology.

[24] England PC, Walker RT, Fu B, Floyd MA (2013). A bound on the viscosity of the Tibetan crust from the horizontality of paleo lake shorelines. Earth Planet Sci Lett..

[25] Yin, A. & Taylor, M. H. Mechanics of V-shaped conjugate strike-slip faults and the corresponding continuum mode of continental deformation. 10.1130/B30159.1 (2011).

[26] Rippe D, Unsworth M (2010). Quantifying crustal flow in Tibet with magnetotelluric data. Phys Earth Planet Int..

[27] Cook K, Royden LH (2008). The role of crustal strength variations in shaping orogenic plateaus, with application to Tibet. J Geophys Res..

[28] Beaumont C (2001). Himalayan tectonics explained extrusions of low viscosity crustal channel coupled to focused surface denudation. Nature.

[29] Clark KM, Bush JWM, Royden LH (2005). Dynamic topography produced by lower crustal flow against rheological strength heterogeneities bordering the Tibetan Plateau. Geophys J Int..

[30] Clark MK, Royden LH (2000). Topographic ooze: Building the eastern margin of Tibet by lower crustal flow. Geology.

[31] Munt I-J, Platt JP (2006). Influence of mantle dynamics on the topographic evolution of the Tibetan Plateau: Result numerical modelling. Tectonics.

[32] Yang Y, Liu M (2008). Crustal thickening and lateral extrusion during the Indo-Asian collision: A 3D viscous flow model. Tectonophysics.

[33] Copley A, Avouac J-P, Wernicke BP (2011). Evidence for mechanical coupling and strong Indian lower crust beneath southern Tibet. Nature.

[34] Banerjee, P., Bürgmann, R., Nagarajan, B. & Apel, E. Intraplate deformation of the Indian subcontinent. *Geophys Res Lett*. **35**, 10.1029/2008GL035468 (2008).

[35] Schiffman C, Bali BS, Szeliga W, Bilham R (2013). Seismic slip deficit in the Kashmir Himalaya from GPS observations. Geophys Res Lett..

[36] Marechal A (2016). Evidence of interseismic coupling variations along the Bhutan Himalayan arc from new GPS data. Geophys Res Lett..

[37] Vernant, P. *et al* Clockwise rotation of the Brahmaputra Valley relative to India: Tectonic convergence in the eastern Himalaya, Naga Hills, and Shillong Plateau. *J Geophys Res Solid Earth* **119**, 10.1002/2014JB011196 (2014).

[38] Devachandra M, Kundu B, Catherine J, Kumar A, Gahalaut VK (2014). Global Positioning System (GPS) Measurements of Crustal Deformation across the Frontal Eastern Himalayan Syntaxis and Seismic-Hazard Assessment. Bull Seismol Soc Am..

[39] Nocquet J-M (2012). Present-day kinematics of the Mediterranean: A comprehensive overview of GPS results. Tectonophysics.

[40] Zhang P-Z (2004). Continuous deformation of the Tibetan Plateau from global positioning system data. Geology.

[41] Shen Z-K, Lu JN, Wang M, Bürgmann R (2005). Contemporary crustal deformation around the southeast borderland of the Tibetan Plateau. J Geophys Res..

[42] Maurin T, Masson F, Rangin C, Than Min U, Collard P (2010). First global positioning system results in northern Myanmar: Constant and localized slip rate along the Sagaing fault. Geology.

[43] Gahalaut VK (2013). Aseismic plate boundary in the Indo-Burmese wedge, northwest Sunda arc. Geology.

[44] Steckler MS (2016). Locked and loading megathrust linked to active subduction beneath the Indo-Burman Ranges. Nat Geosci..

[45] Altamimi Z, Collilieux X, Métivier (2011). L. ITRF2008: An improved solution of the International Terrestrial Reference Frame. J. Geod..

[46] Okada Y (1992). Internal deformation due to shear and tensile faults in a half-space. Bull Seismol Soc Am..

[47] Angelier J, Baruah S (2009). Seismotectonics in Northeast India: a stress analysis of focal mechanism solutions of earthquakes and its kinematic implications. Geophys J Int..

[48] Ravi Kumar, M. *et al* Shear wave anisotropy in the Eastern Himalaya, Burmese arc and adjoining regions, AGU abstract in Advances in Understanding Earth’s Dynamic Processes using Seismic Anisotropy (2017).

[49] Maurin T, Rangin C (2009). Structure and kinematics of the Indo-Burmese Wedge: Recent and fast growth of the outer wedge. Tectonics.

[50] Najman Y, Bracciali L, Parrish RR, Chisty E, Copley A (2015). Evolving strain partitioning in the Eastern Himalaya: The growth of the Shillong Plateau. Earth Planet Sci Lett..

[51] Passchier CW, Platt J (2016). Shear zone junctions: Of zippers and freeways. Journal of Structural Geology.

[52] Platt J, Passchier CW (2016). Zipper junctions: A new approach to the intersections of conjugate strike-slip faults. Geology.

[53] Fitch TJ (1972). Plate convergence, transcurrent faults, and internal deformation adjacent to Southeast Asia and the western Pacific. J Geophys Res..

[54] Jarrard RD (1986). Terrane Motion by Strike-slip Faulting of Fore-arc Systems. Geology.

[55] Nacquet J-M (2014). Motion of continental slivers and creeping subduction in the northern Andes. Nat Geosci..

[56] Ishii M, Kiser E, Geist EL (2013). Mw 8.6 Sumatran earthquake of 11 April 2012: Rare seaward expression of oblique subduction. Geology.

[57] Hammer P (2013). Flexure of the India plate underneath the Bhutan Himalaya. Geophys Res Lett..

[58] Turcotte, D. & Schubert, G. Geodynamics; applications of continuum physics to geological problems. New York, John Wiley & Sons, 450 (1982).

[59] Bilham R, Mencin D, Bendick R, Burgmann R (2017). Implications for elastic energy storage in the Himalaya from the Gorkha 2015 earthquake and other incomplete ruptures of the Main Himalayan Thrust. Quaternary International.

