# Supplementary material for: Oblique convergence and strain partitioning in the outer deformation front of NE Himalaya
Source: Sci Rep. 2018 Jul 12;8:10564. doi: 10.1038/s41598-018-28774-3 (PMC6043504; doi:10.1038/s41598-018-28774-3)
Supplement: Supplementary file 1 — Supplementary Information [file 41598_2018_28774_MOESM1_ESM.docx]

**SUPPLEMENTARY MATERIAL**

Oblique convergence and strain partitioning in the outer deformation front of NE Himalaya

**Dibyashakti Panda^1^, Bhaskar Kundu^1*^, M Santosh^2,3,4^**

^1^Department of Earth and Atmospheric Sciences, NIT Rourkela, Rourkela-769008, India

^2^School of Earth Science and Resources, China University of Geosciences, Beijing, China

^3^Department of Earth Sciences, University of Adelaide, SA 5005, Australia

^4^State Key Laboratory of Continental Dynamics, Department of Geology, Northwest University, Xi’an 710069, China

**^*^Corresponding author:** Bhaskar Kundu, Department of Earth and Atmospheric Sciences, NIT Rourkela, Rourkela-769008, India, ([rilbhaskar@gmail.com](mailto:rilbhaskar@gmail.com)).

| **Structures** | **Strike (**$\boldsymbol{^{\circ}}$**)** | **Dip (**$\boldsymbol{^{\circ}}$**)** | **Locking Depth (km)** | **Fault Parallel motion (mm/yr)** | **Fault Normal motion (mm/yr)** |
| --- | --- | --- | --- | --- | --- |
| **MFT:** Main Frontal Thrust (Bhutan Himalaya) | 270 | 7$\pm2$ | 92$\pm5$ | -- | 11.5$\pm2$ |
| **MFT:** Main Frontal Thrust (Arunachal Himalaya) | 230 | 7±2 | 112$\pm10$ | -- | 16$\pm3$ |
| **DF:** Dauki Fault | 270 | 30 | 20$\pm5$ | -- | 8$\pm2$ |
| **NT:** Naga Thrust | 50 | 23 | 30$\pm5$ | 7$\pm2$ | -- |

**Table 1.** Fault geometry and predicted fault parallel/normal motion from GPS observations.
